# Sustained disease control with aflibercept 8 mg: a new benchmark in the management of retinal neovascular diseases

**DOI:** 10.1038/s41433-024-03312-w

**Published:** 2024-08-31

**Authors:** Jean-François Korobelnik, Paolo Lanzetta, Charles C. Wykoff, Tien Y. Wong, Xin Zhang, Peter Morgan-Warren, Scott Fitzpatrick, Sergio Leal, Lynne Brunck, Zoran Hasanbasic, Karen W. Chu, Kimberly Reed, Sobha Sivaprasad

**Affiliations:** 1 CHU Bordeaux, Service d’ophtalmologie, Bordeaux, France; 2 University of Bordeaux, INSERM, BPH, UMR1219, Bordeaux, France; 3 Department of Medicine - Ophthalmology, University of Udine, and Istituto Europeo di Microchirurgia Oculare - IEMO, Udine, Italy; 4 Retina Consultants of Texas, Retina Consultants of America, Blanton Eye Institute, Houston Methodist Hospital, Houston, TX USA; 5 Singapore Eye Research Institute, Singapore National Eye Centre, Singapore, Singapore; 6 Tsinghua Medicine, Tsinghua University, Beijing, China; 7 Bayer Consumer Care AG, Basel, Switzerland; 8 Bayer Inc., Mississauga, Ontario Canada; 9 Regeneron Pharmaceuticals, Inc., Tarrytown, NY USA; 10 NIHR Moorfields Biomedical Research Centre, Moorfields Eye Hospital NHS Foundation Trust, London, UK

**Keywords:** Retinal diseases, Quality of life

Retinal neovascular diseases, such as neovascular age-related macular degeneration (nAMD), diabetic retinopathy (DR), diabetic macular oedema (DMO), and retinal vein occlusion (RVO), are major causes of visual impairment worldwide [1–3]. Vascular endothelial growth factor (VEGF) is considered critical in the pathophysiology of these conditions and randomized trials have established the efficacy and safety of agents with an anti-VEGF mechanism of action [4]. Extensive real-world studies of ranibizumab or aflibercept 2 mg have also demonstrated that effectiveness in clinical practice is possible [5–8], including maintenance of vision gains through 4 years with a proactive treat-and-extend (T&E) regimen in patients with nAMD [5], and vision gains over 3 years with a meaningful reduction in treatment burden after early, consistent dosing in patients with DMO [6].

Patients, physicians, and caregivers generally prefer regimens with fewer clinic visits, and although T&E regimens allow gradual treatment interval extensions once disease inactivity is verified, treatment burden to achieve optimal outcomes can remain high [9, 10]. Consequently, many patients in real-life settings receive fewer anti-VEGF injections than administered in clinical trials, despite dosing recommendations in the regulatory-approved product label, with potentially fewer monitoring visits often resulting in suboptimal outcomes [11], leading to non-adherence and non-persistence to long-term therapy [12].

To address these real-world challenges, we introduce a new concept of “sustained disease control”, which is characterized by three inter-related goals of maintaining vision gains, rapid and resilient fluid control without clinically meaningful fluctuation, and with extended treatment intervals. We suggest this will address the treatment burden associated with frequent injections and/or monitoring visits, while maintaining the benefits of anti-VEGF therapy. The inter-relationship between these three factors is particularly evident in nAMD, where restoration and consistent stabilization of retinal fluid supports achievement and maintenance of vision gains and allows treatment interval extensions, while unchecked fluid recurrence can lead to suboptimal visual outcomes [13].

Previous studies have demonstrated that increased anti-VEGF doses may result in improved anatomic outcomes (aflibercept 0.5 to 4 mg) [14] or increased durability (ranibizumab 0.5 to 2 mg) [15]. Recently, newer treatment options for nAMD (brolucizumab, faricimab and aflibercept 8 mg) have demonstrated non-inferior vision gains versus aflibercept 2 mg (the standard of care) [16–18]. More specifically, increasing the aflibercept dose from 2 mg to 8 mg aims to reduce treatment burden by providing a 4-fold higher molar dose, hypothesized to prolong ocular VEGF inhibition [19]. The available evidence on functional and anatomic outcomes and extended treatment intervals suggests that aflibercept 8 mg is able to deliver sustained disease control in a substantial proportion of patients (Table 1) [18, 20].

**Table 1:** Clinical trial evidence for aflibercept 8 mg in nAMD and DME.

Category	Evidence
Vision gains maintained	**PULSAR**:
	• Primary endpoint: LS mean change from baseline in BCVA letter score at Week 48 was +6.7 in the aflibercept 8q12 group, +6.2 in the 8q16 group, and +7.6 in the aflibercept 2q8 group. The estimated difference in LS mean changes (95% CI; confirmatory non-inferiority test at 4-letter margin) from baseline to Week 48 in BCVA letter score for aflibercept 8q12 vs 2q8 was –0.97 (–2.87 to 0.92; *p* = 0.0009) and for aflibercept 8q16 vs 2q8 was –1.14 (–2.97 to 0.69; *p* = 0.0011) [18].
	• LS mean change from baseline in BCVA letter score at Week 96 was +5.6 in the aflibercept 8q12 group, +5.5 in the 8q16 group, and +6.6 in the aflibercept 2q8 group. The estimated difference in LS mean changes (95% CI; nominal non-inferiority test at 4-letter margin) from baseline to Week 96 in BCVA letter score for aflibercept 8q12 vs 2q8 was –1.01 (–2.82 to 0.80; *p* = 0.0006 [nominal]) and for aflibercept 8q16 vs 2q8 was –1.08 (–2.87 to 0.71; *p* = 0.0007 [nominal]) [24].
	**PHOTON**:
	• Primary endpoint: LS mean change from baseline in BCVA letter score at Week 48 was +8.8 in the aflibercept 8q12 group, +7.9 in the 8q16 group, and +9.2 in the aflibercept 2q8 group. The estimated difference in LS mean changes (95% CI; confirmatory non-inferiority test at 4-letter margin) from baseline to Week 48 in BCVA letter score for 8q12 vs 2q8 was –0.57 (–2.26 to 1.13; *p* < 0.0001) and for aflibercept 8q16 vs 2q8 –1.44 (–3.27 to 0.39; *p* = 0.0031) [20].
	• LS mean change from baseline in BCVA letter score at Week 96 was +8.2 in the aflibercept 8q12 group, +6.6 in the 8q16 group, and +7.7 in the aflibercept 2q8 group. The estimated difference in LS mean changes (95% CI; nominal non-inferiority test at 4-letter margin) from baseline to Week 96 in BCVA letter score for aflibercept 8q12 vs 2q8 was +0.5 (–1.6 to 2.5; *p* < 0.0001 [nominal]) and for aflibercept 8q16 vs 2q8 was –1.1 (–3.3 to 1.1; *p* = 0.0044 [nominal]) [23].
Anatomic results	**PULSAR**:
	• Aflibercept 8 mg demonstrated a numerically faster median time to fluid-free central subfield vs aflibercept 2 mg (4 weeks with each aflibercept 8q12 and 8q16 groups vs 8 weeks with aflibercept 2 mg) [25].
	• Key secondary endpoint: The pooled aflibercept 8 mg group showed statistically significant superiority vs aflibercept 2 mg in the proportion of patients with no retinal fluid in the center subfield at Week 16: 422 (63%) of 667 vs 173 (52%) of 335, respectively, with a treatment difference of 12% (95% CI: 5 to 18%; superiority test *p* = 0.0002) in favor of aflibercept 8 mg [18].
	• Aflibercept 8 mg demonstrated a similar change in CRT to aflibercept 2 mg through Week 96. LS mean change in CRT from baseline to Week 48 was ‒147 μm, ‒147 μm and ‒136 μm in the aflibercept 8q12, 8q16, and 2q8 groups, respectively [18]. At Week 96, mean change in CRT from baseline was ‒152 μm, ‒149 μm, and ‒147 μm in the aflibercept 8q12, 8q16, and 2q8 groups, respectively [24].
	**PHOTON:**
	• Aflibercept 8 mg demonstrated a similar change in CRT to aflibercept 2 mg through Week 96. Mean change in CRT from baseline to Week 48 was ‒171 μm, ‒148 μm, and ‒165 μm in the aflibercept 8q12, 8q16, and 2q8 groups, respectively [20]. At Week 96, mean change in CRT from baseline was ‒185 μm, ‒155 μm, and ‒187 μm for the aflibercept 8q12, 8q16, and 2q8 groups, respectively [23].
Extended treatment intervals	**PULSAR**:
	• Overall, 88%, 71%, and 47% of patients receiving aflibercept 8 mg had assigned ≥12-week, ≥16-week, and ≥20-week dosing intervals at Week 96 [24]. ◦ In the aflibercept 8q16 group, 53% of patients had ≥20-week and 31% had 24-week dosing intervals at Week 96 [24].
	• Patients received ~8 injections in the aflibercept 8q16 group vs ~13 injections in the aflibercept 2q8 group through Week 96 [24].
	**PHOTON**:
	• Overall, 93%, 72%, and 44% of patients receiving aflibercept 8 mg had assigned ≥12-week, ≥16-week, and ≥20-week dosing intervals at Week 96 [23]. ◦ In the aflibercept 8q16 group, 46% of patients had ≥20-week and 32% had 24-week dosing intervals at Week 96 [23].
	• During the initial treatment phase, the aflibercept 8 mg groups received 3 vs 5 initial monthly injections with aflibercept 2 mg [20].
	• Patients received ~8 injections in the aflibercept 8q16 group vs ~14 injections in the aflibercept 2q8 group through Week 96 [23].

2q8, 2 mg every 8 weeks; 8q12, 8 mg every 12 weeks; 8q16, 8 mg every 16 weeks; BCVA, best correct visual acuity; CI, confidence interval; CRT, central subfield retinal thickness; LS, least squares.

Aflibercept 8 mg is approved for the treatment of nAMD, DR, and DMO in various geographies based on robust clinical trial data. Firstly, aflibercept 8 mg was evaluated in the controlled phase 2, 44-week CANDELA study in 106 patients with nAMD where the safety profiles of aflibercept 8 mg and 2 mg, administered using the same dosing schedule (3 monthly injections followed by doses at Weeks 20 and 32, unless additional injections were warranted), were comparable [21]. In that data set, aflibercept 8 mg showed numerically greater anatomic and visual improvements compared with aflibercept 2 mg [21], with more patients without fluid in the central subfield at Week 16 [21]. Further, 3 pivotal trials are investigating the efficacy and safety of extended dosing with aflibercept 8 mg compared with aflibercept 2 mg every 8 weeks (2q8) in nAMD and DMO, or aflibercept 2 mg every 4 weeks in RVO [18, 20, 22]. QUASAR (NCT05850520) is a currently underway phase 3 study in patients with macular oedema secondary to RVO [22], while patients in the phase 3 study in nAMD (PULSAR: NCT04423718) and phase 2/3 study in DMO (PHOTON: NCT04429503) have completed masked 96-week treatment and are in the open-label extension phase [17, 20].

PULSAR and PHOTON used an algorithm in which treatment intervals could be modified if protocol-defined dose regimen modification (DRM) criteria were met [18, 20]. The dosing intervals for patients in the groups receiving aflibercept 8 mg every 12 (8q12) or 16 weeks (8q16) could be shortened from Week 16 (if DRM criteria denoting disease activity were met at any dosing visit; minimum dosing interval of 8 weeks) and extended from Week 52 (if both functional and anatomic DRM stability criteria compared with Week 12 were met) [18, 20].

In both PULSAR and PHOTON, the primary endpoint of non-inferior visual gains with aflibercept 8 mg versus aflibercept 2 mg at Week 48 were met, with visual acuity improvements maintained through 96 weeks [23, 24]. The similar vision gains were achieved and maintained over 96 weeks with ~8 injections in the aflibercept 8 mg arms of both studies and ~13 (PULSAR) or ~14 (PHOTON) injections in the aflibercept 2 mg arm [18, 20]. Overall, 53% and 47% of patients initially randomized to aflibercept 8q16 in PULSAR and PHOTON, respectively, had last assigned treatment intervals of at least 20 weeks [23, 24]. In PULSAR, aflibercept 8 mg demonstrated superior drying compared with aflibercept 2 mg at Week 16, a key secondary endpoint for the trial [18], as well as rapid fluid control, with a numerically faster median time to fluid-free central subfield versus aflibercept 2 mg and fluid control maintained at Week 96 [18, 24, 25]. In both PULSAR and PHOTON, aflibercept 8 mg demonstrated a similar change in central retinal thickness to aflibercept 2 mg at Week 96 [23, 24]. Finally, the safety results of aflibercept 8 mg were comparable to the well-established safety profile of aflibercept 2 mg [23, 24].

Taken together, these findings provide support that aflibercept 8 mg can deliver sustained disease control in a substantial proportion of patients with nAMD or DMO through 96 weeks, with extended treatment intervals [18, 20]. While some patients may still require more frequent treatment, aflibercept 8 mg offers an opportunity for clinicians to meaningfully extend treatment intervals for most patients while maintaining vision gains and fluid control.

## Data Availability

All data generated or analysed during this study are included in this published article.

## References

[1] Mitchell P, Liew G, Gopinath B, Wong TY. Age-related macular degeneration. Lancet. 2018;392:1147–59. 30303083 10.1016/S0140-6736(18)31550-2

[2] Wong TY, Cheung CM, Larsen M, Sharma S, Simo R. Diabetic retinopathy. Nat Rev Dis Prim. 2016;2:16012. 27159554 10.1038/nrdp.2016.12

[3] Nicholson L, Talks SJ, Amoaku W, Talks K, Sivaprasad S. Retinal vein occlusion (RVO) guideline: executive summary. Eye. 2022;36:909–12. 35301458 10.1038/s41433-022-02007-4 PMC9046155

[4] Tan CS, Ngo WK, Chay IW, Ting DS, Sadda SR. Neovascular age-related macular degeneration (nAMD): a review of emerging treatment options. Clin Ophthalmol. 2022;16:917–33. 35368240 10.2147/OPTH.S231913 PMC8965014

[5] Traine PG, Pfister IB, Zandi S, Spindler J, Garweg JG. Long-term outcome of intravitreal aflibercept treatment for neovascular age-related macular degeneration using a “treat-and-extend” regimen. Ophthalmol Retin. 2019;3:393–9. 10.1016/j.oret.2019.01.018 31044729

[6] Lukic M, Williams G, Shalchi Z, Patel PJ, Hykin PG, Hamilton RD, et al. Intravitreal aflibercept for diabetic macular oedema in real-world: 36-month visual acuity and anatomical outcomes. Eur J Ophthalmol. 2021;31:1201–7. 32429690 10.1177/1120672120925034

[7] Holz FG, Figueroa MS, Bandello F, Yang Y, Ohji M, Dai H, et al. RANIBIZUMAB treatment in treatment-naive neovascular age-related macular degeneration: results from luminous, a Global real-world study. Retina. 2020;40:1673–85. 31764612 10.1097/IAE.0000000000002670 PMC7447127

[8] Ashraf M, Souka AAR. Aflibercept in age-related macular degeneration: evaluating its role as a primary therapeutic option. Eye. 2017;31:1523–36. 28548650 10.1038/eye.2017.81 PMC5684459

[9] Loewenstein A, Sylvanowicz M, Amoaku WM, Aslam T, Cheung GCM, Eldem B et al. A global survey of patients, providers, and clinic staff from 24 countries reveals barriers to optimal care delivery for nAMD due to constraints and gaps in clinic capacity. Presented at EURETINA 2023. Available at: https://euretina.softr.app/amsterdam-abstract?recordId=rec6swwKES5yLhHNn. Accessed January 2024.

[10] Ziemssen F, Sylvanowicz M, Amoaku wm, Aslam T, Eldem B, Finger R et al. Opportunities to improve clinical management of diabetic retinopathy and diabetic macular edema: Insights from global survey data of patients, providers, and clinic staff from 24 countries. Presented at EURETINA 2023. Available at: https://euretina.softr.app/amsterdam-abstract?recordId=recmlKzQAcZpAXGP0. Accessed January 2024.

[11] Ehlken C, Helms M, Bohringer D, Agostini HT, Stahl A. Association of treatment adherence with real-life VA outcomes in AMD, DME, and BRVO patients. Clin Ophthalmol. 2018;12:13–20. 29339917 10.2147/OPTH.S151611 PMC5745150

[12] Okada M, Mitchell P, Finger RP, Eldem B, Talks SJ, Hirst C, et al. Nonadherence or nonpersistence to intravitreal injection therapy for neovascular age-related macular degeneration: a mixed-methods systematic review. Ophthalmology. 2021;128:234–47. 32763265 10.1016/j.ophtha.2020.07.060 PMC7403101

[13] Chakravarthy U, Havilio M, Syntosi A, Pillai N, Wilkes E, Benyamini G, et al. Impact of macular fluid volume fluctuations on visual acuity during anti-VEGF therapy in eyes with nAMD. Eye. 2021;35:2983–90. 33414525 10.1038/s41433-020-01354-4 PMC8526705

[14] Heier JS, Boyer D, Nguyen QD, Marcus D, Roth DB, Yancopoulos G, et al. The 1-year results of CLEAR-IT 2, a phase 2 study of vascular endothelial growth factor trap-eye dosed as-needed after 12-week fixed dosing. Ophthalmology. 2011;118:1098–106. 21640258 10.1016/j.ophtha.2011.03.020

[15] Ho AC, Busbee BG, Regillo CD, Wieland MR, Van Everen SA, Li Z, et al. Twenty-four-month efficacy and safety of 0.5 mg or 2.0 mg ranibizumab in patients with subfoveal neovascular age-related macular degeneration. Ophthalmology. 2014;121:2181–92. 25015215 10.1016/j.ophtha.2014.05.009

[16] Dugel PU, Koh A, Ogura Y, Jaffe GJ, Schmidt-Erfurth U, Brown DM, et al. HAWK and HARRIER: Phase 3, multicenter, randomized, double-masked trials of Brolucizumab for neovascular age-related macular degeneration. Ophthalmology. 2020;127:72–84. 30986442 10.1016/j.ophtha.2019.04.017

[17] Heier JS, Khanani AM, Quezada Ruiz C, Basu K, Ferrone PJ, Brittain C, et al. Efficacy, durability, and safety of intravitreal faricimab up to every 16 weeks for neovascular age-related macular degeneration (TENAYA and LUCERNE): two randomised, double-masked, phase 3, non-inferiority trials. Lancet. 2022;399:729–40. 35085502 10.1016/S0140-6736(22)00010-1

[18] Lanzetta P, Korobelnik JF, Heier JS, Leal S, Holz FG, Clark WL, et al. Intravitreal aflibercept 8 mg in neovascular age-related macular degeneration (PULSAR): 48-week results from a randomised, double-masked, non-inferiority, phase 3 trial. Lancet. 2024;403:1141–52. 10.1016/S0140-6736(24)00063-1 38461841

[19] Veritti D, Sarao V, Di Bin F, Lanzetta P. Pharmacokinetic and pharmacodynamic rationale for extending VEGF inhibition increasing intravitreal aflibercept dose. Pharmaceutics. 2023;15:1416. 37242658 10.3390/pharmaceutics15051416 PMC10220677

[20] Brown DM, Boyer DS, Do DV, Wykoff CC, Sakamoto T, Win P, et al. Intravitreal aflibercept 8 mg in diabetic macular oedema (PHOTON): 48-week results from a randomised, double-masked, non-inferiority, phase 2/3 trial. Lancet. 2024;403:1153–63. 10.1016/S0140-6736(23)02577-1 38461843

[21] Wykoff CC, Brown DM, Reed K, Berliner AJ, Gerstenblith AT, Breazna A, et al. Effect of high-dose intravitreal aflibercept, 8 mg, in patients with neovascular age-related macular degeneration: the Phase 2 CANDELA randomized clinical trial. JAMA Ophthalmol. 2023;141:834–42. 37535382 10.1001/jamaophthalmol.2023.2421 PMC12278850

[22] ClinicalTrials.gov. A study to learn how well a higher amount of aflibercept given as an injection into the eye works and how safe it is in people with reduced vision due to swelling in the macula, central part of the retina caused by a blocked vein in the retina (macula edema secondary to retinal vein occlusion) (QUASAR). Available at: https://classic.clinicaltrials.gov/ct2/show/NCT05850520. Accessed January 2024.

[23] Do DV. Aflibercept 8 mg for diabetic macular edema: 2-year results of the phase 2/3 PHOTON trial. Presented at American Society of Retina Specialists 2023 Meeting. Available at: https://investor.regeneron.com/static-files/c5aea914-bada-4d6f-8742-817e319b972f. Accessed January 2024.

[24] Lanzetta P, Schulze A, Schmidt-Ott U, Zhang X, Berliner A, Chu K et al. Intravitreal aflibercept 8 mg injection in patients with neovascular age-related macular degeneration: 60-week and 96-week results from the phase 3 PULSAR trial. Presented at the 23rd European Society of Retina Specialists (EURETINA) Congress. Available at: https://investor.regeneron.com/static-files/06016223-ff10-4fff-b9bb-3162f0ef27d9. Accessed January 2024.

[25] Korobelnik J-F. Intravitreal aflibercept 8 mg injection in patients with neovascular age-related macular degeneration: 48-week results from the phase 3 PULSAR trial. Presented at the Retina Society 55th Annual Scientific Meeting. Available at: https://investor.regeneron.com/static-files/d2cb2519-182f-42a6-8ac4-04544883febc. Accessed January 2024.

